# Supplementary material for: Foveal vision anticipates defining features of eye movement targets
Source: eLife. 2022 Sep 9;11:e78106. doi: 10.7554/eLife.78106 (PMC9581528; doi:10.7554/eLife.78106)
Supplement: Transparent reporting form [file elife-78106-transrepform1.docx]

***eLife’s* transparent reporting form**

We encourage authors to provide detailed information *within their submission* to facilitate the interpretation and replication of experiments. Authors can upload supporting documentation to indicate the use of appropriate reporting guidelines for health-related research (see [EQUATOR Network](http://www.equator-network.org/%20)), life science research (see the [BioSharing Information Resource](https://biosharing.org/)), or the [ARRIVE guidelines](http://www.plosbiology.org/article/info:doi/10.1371/journal.pbio.1000412) for reporting work involving animal research. Where applicable, authors should refer to any relevant reporting standards documents in this form.

If you have any questions, please consult our Journal Policies and/or contact us: [editorial@elifesciences.org](mailto:editorial@elifesciences.org).

**Sample-size estimation**

- You should state whether an appropriate sample size was computed when the study was being designed
- You should state the statistical method of sample size computation and any required assumptions
- If no explicit power analysis was used, you should describe how you decided what sample (replicate) size (number) to use

Please outline where this information can be found within the submission (e.g., sections or figure legends), or explain why this information doesn’t apply to your submission:

We state our sample size rationale in the section ‘Sample Size Rationale’ in the Supplemental Materials.

**Replicates**

- You should report how often each experiment was performed
- You should include a definition of biological versus technical replication
- The data obtained should be provided and sufficient information should be provided to indicate the number of independent biological and/or technical replicates
- If you encountered any outliers, you should describe how these were handled
- Criteria for exclusion/inclusion of data should be clearly stated
- High-throughput sequence data should be uploaded before submission, with a private link for reviewers provided (these are available from both GEO and ArrayExpress)

Please outline where this information can be found within the submission (e.g., sections or figure legends), or explain why this information doesn’t apply to your submission:

Each experiment was performed once.

The data and experimental code are available here:

https://osf.io/v9gsq/

The links for all repositories are provided in the ‘Sample’ section in the Methods. In the same section, we explicitly state how many observers who had completed Experiment 1 also completed Experiments 2 and 3, respectively (biological replication). In Figure 5, we connect the data points of observers who participated in both experiments.
We did not exclude any outliers.

We explicitly list all trial exclusion criteria applied during the pre-processing of eye movement data in the section Methods – Data analysis – Eye movement pre-processing.

We did not collect high-throughput sequence data.

**Statistical reporting**

- Statistical analysis methods should be described and justified
- Raw data should be presented in figures whenever informative to do so (typically when N per group is less than 10)
- For each experiment, you should identify the statistical tests used, exact values of N, definitions of center, methods of multiple test correction, and dispersion and precision measures (e.g., mean, median, SD, SEM, confidence intervals; and, for the major substantive results, a measure of effect size (e.g., Pearson's r, Cohen's d)
- Report exact p-values wherever possible alongside the summary statistics and 95% confidence intervals. These should be reported for all key questions and not only when the p-value is less than 0.05.

Please outline where this information can be found within the submission (e.g., sections or figure legends), or explain why this information doesn’t apply to your submission:

All statistical analysis methods are described in the section Methods – Data analysis. When applicable, we provide further details for those analyses in the Supplemental Materials – Supplemental Methods.

Our dataset constitutes a ‘small-n’ design, meaning that we collected a large number of trials in a small number of participants. Each experiment included 7-9 observers. Since each observer contributed several thousands of trials, the results presented in this manuscript include approximately 57,000 trials. Nonetheless, we provide individual observer data in the Supplements (Figure S1) and plot observers as individual data points in Figure 5.

In the Results section, we report exact p-values along with summary statistics. Whenever our conclusions rely on a null-effect, we additionally provide Bayes factors.

(For large datasets, or papers with a very large number of statistical tests, you may upload a single table file with tests, Ns, etc., with reference to sections in the manuscript.)

**Group allocation**

- Indicate how samples were allocated into experimental groups (in the case of clinical studies, please specify allocation to treatment method); if randomization was used, please also state if restricted randomization was applied
- Indicate if masking was used during group allocation, data collection and/or data analysis

Please outline where this information can be found within the submission (e.g., sections or figure legends), or explain why this information doesn’t apply to your submission:

We did not assign observers to groups or used any kind of masking.

**Additional data files (“source data”)**

- We encourage you to upload relevant additional data files, such as numerical data that are represented as a graph in a figure, or as a summary table
- Where provided, these should be in the most useful format, and they can be uploaded as “Source data” files linked to a main figure or table
- Include model definition files including the full list of parameters used
- Include code used for data analysis (e.g., R, MatLab)
- Avoid stating that data files are “available upon request”

Please indicate the figures or tables for which source data files have been provided:

The experimental code, eye-movement and response data as well as stimulus materials are available here: https://osf.io/v9gsq/
